# Distinct promoter methylation patterns of *LKB1* in the hamartomatous polyps of Peutz-Jeghers syndrome and its potential in gastrointestinal malignancy prediction

**DOI:** 10.1186/s13023-020-01502-9

**Published:** 2020-08-15

**Authors:** Teng Li, Wensheng Lin, Yilei Zhao, Jianping Zhu, Tao Sun, Li Ren

**Affiliations:** 1 Department of Pathology, Air Force Medical Center, PLA, Beijing, China; 2 Department of Pathology, Huizhou Third People’s Hospital, Guangzhou Medical University, Huizhou, China; 3 Department of Gastroenterology, Air Force Medical Center, PLA, Beijing, China

**Keywords:** DNA methylation, Peutz-Jeghers syndrome, Liver kinase B1, Hamartomatous polyp, Colorectal Cancer, Prognosis

## Abstract

**Background:**

Peutz-Jeghers Syndrome (PJS) is known as a rare inherited polyposis due to the malfunction of serine/threonine kinase gene *LKB1*. However, not all of PJS patients carry *LKB1* germline mutation. Previous researches have observed the elevated DNA methylation level in PJS polyps. Nevertheless, the mechanism of such abnormal and its impact on PJS patients remains to be fully described.

**Results:**

The results proved a significant increase on the methylation level of *LKB1* promoter in PJS polyps compared with normal colon biopsies through bisulfite PCR followed by Sanger sequencing. Moreover, the methylation pattern in PJS polyps could be further categorized as three different scenarios: hypermethylated, hemimethylated and hypomethylated pattern. Furthermore, immunohistochemistry of DNMT1/3a/3b suggested the up-regulation of DNMT1 and 3a might participate the epigenetic alternation of *LKB1* in PJS polyps. Logistic regression suggested hypomethylated *LKB1* promoter in PJS polyps as a risk factor for gastrointestinal malignancies in PJS patients.

**Conclusions:**

The promoter methylation level of *LKB1* gene in PJS polyps is generally elevated compared with normal colon mucosa. Yet not all of PJS polyps carry hypermethylated *LKB1* promoter. Hypomethylation in this region has linked to malignant tumors in PJS patients. Given the rarity of PJS, this work together with previous researches, have proved the importance of *LKB1* promoter methylation in PJS development and prognosis.

## Introduction

Peutz-Jeghers syndrome (PJS) is a rare disease due to the malfunction of *LKB1* (*STK11*) gene [1]. The clinical pathological features to diagnose PJS include: gastrointestinal harmartomatous polyps, mucocutanenous pigmentation and family history [2]. PJS could be lethal for the polyp-related complications, especially intussusceptions, and for the substantial risk (up to 86% of life-time accumulation risk) of adenocarcinoma in the gastrointestinal tract in such patients [3]. Moreover, PJS could harm childhood healthiness, as many PJS patients developed obstruction and intussusception before the age of twenty [4], and those symptoms could be found as early as 4-year old (according to our center’s experience). Double balloon pushed enteroscopy (DBE) surveillance have been proved to help PJS patients by detection and removal of polyps and the consequent referral of selected patients for surgery [5]. The department of gastroenterology of our center is one of the DBE centers in China. Thus, we have collected polyp samples from more than 300 PJS patients.

Although previous researches have proved the majority of PJS patients carry *LKB1* exon mutation [6–9], others suggested *LKB1* mutation might not be the only explanation [10]. In 2000, researchers use methylation specific PCR (MSP) method first detected aberrant DNA methylation in PJS patients [11]. Following articles suggested the altered *CSX* gene DNA methylation patterns in “normal” epithelial crypt of PJS patients [12]. All these data indicated the involvement of DNA methylation in PJS development. However, due to the rarity of PJS and the method limits, the relationship between *LKB1* promoter methylation and PJS remains to be described. In this study, we use bisulfite PCR followed by Sanger sequencing to determine the methylation status of 21 CpGs in the promoter of *LKB1* gene in 50 PJS polyps and 50 normal colon mucosa. To author’s best knowledge, this is the largest dataset for the characterization of DNA methylation in PJS polyps.

## Results

### Elevated overall methylation level of *LKB1* promoter in PJS polyps

**Fig. 1:**
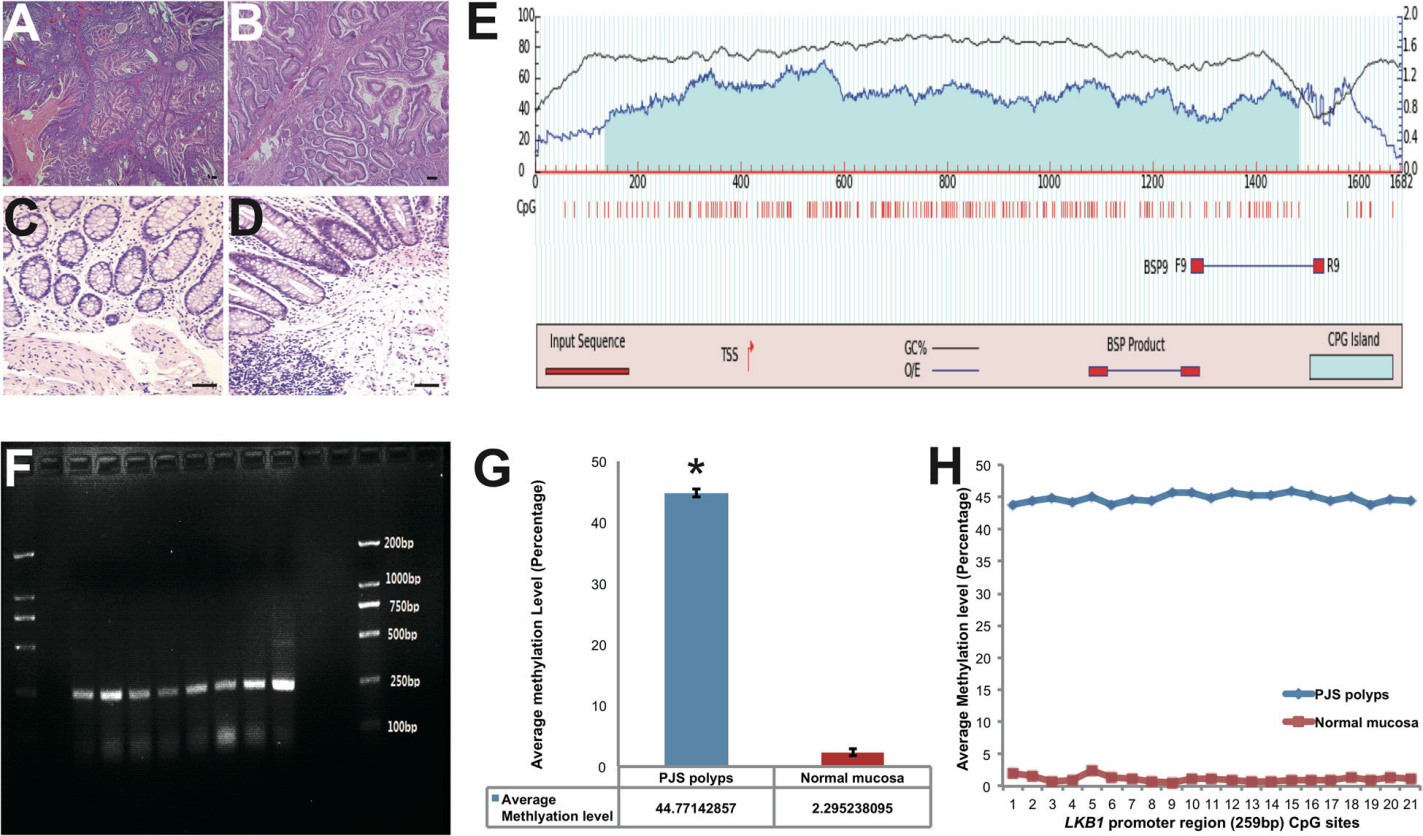
Bisulfite PCR-Sanger sequencing revealed elevated methylation level in the hamartomatous polyps of PJS patients compared with normal mucosa. **a**, **b** Histology of PJS polyp samples used in this study**,** magnificatio*n* = 100x, *n* = 50 **c**, **d** Histology of normal colon mucosa used in this study, magnification = 100x, *n* = 50 **e** Bisulfite PCR Primer design from *LKB1* promoter by MethPrimer. **f** Representative of gel image after bisufite PCR amplifications. The PCR product is 259 bp, *n* = 100 (**g**) Average methylation level for *LKB1* promoter region, comparison between 50 PJS polyps and 50 normal mucosa samples revealed the gap between two groups. Means ± SEM, *P < 0.05. **h** The methylation analysis per each CpG site indicated that instead of randomly distributed, DNA methylation was evenly distributed across the whole region. Data presented as means. All bars = 100 μm

**Fig. 2:**
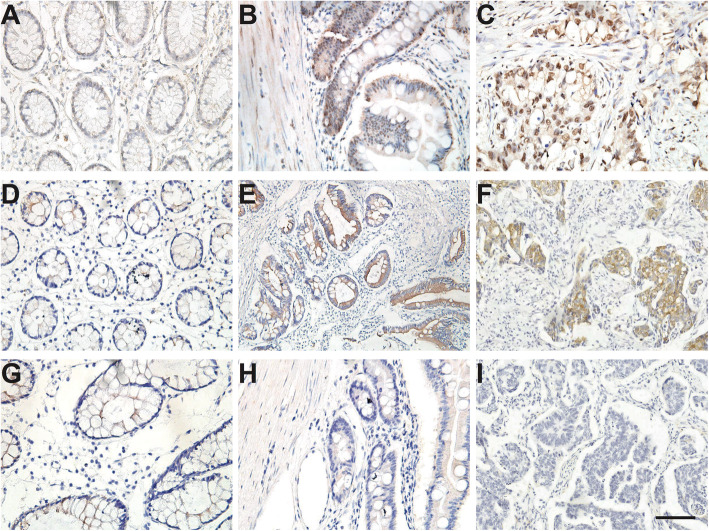
Characterization of DNMTs’ expression in normal colon mucosa, PJS polyps, and colorectal cancer of PJS patients. **a**-**c** DNMT1 expression in the above samples. The expression of DNMT1 is increased in PJS polyps and colorectal cancer in PJS patients compared with normal mucosa. *n* = 15 (**d**-**f**) DNMT3a expression in the above samples. The expression of DNMT3a is increased in PJS polyps and colorectal cancer in PJS patients compared with normal mucosa. *n* = 15 (**g**-**i**) DNMT3b immunochemistry results shows negative staining in all the above samples. *n* = 15. Bar = 100 μm

All the PJS polyps and normal mucosa diagnoses were consensus-decisions by three independent pathologists under HE staining (Fig. 1a-d). In order to explore the overall methylation level of *LKB1* promoter in PJS polyps and normal mucosa, first we analyzed the promoter region of *LKB1* gene and design primers. As shown in Fig. 1e, we selected the core promoter region from the predicted CpG island and designed the bisulfite PCR primer. The PCR product was 259 bp, including 21 CpGs from *LKB1* core promoter (Fig. 1f). The sequencing results indicated, the overall methylation level for the whole region was significantly higher in PJS group than in normal group (Fig. 1h). However, for each CpG site, the methylation level in both PJS and normal group are similar (Fig. 2g).

### Up-regulation of DNMT1 and 3a expression in PJS polyps

To explore the mechanism of how *LKB1* promoter methylation is elevated, we characterized the expression of three DNMTs, i.e. DNMT1, 3a and 3b, in normal colon mucosa, PJS polyps and colorectal cancer in PJS patients. As shown in Fig. 2a-c, DNMT1 is weakly expressed in normal mucosa, while its expression has elevated in the epithelial cells of PJS polyps and colorectal cancer in PJS patients. Similar to DNMT1, DNMT3a also have strong staining in the epithelial cells of PJS polyps and colorectal cancer in PJS patients compared to the normal samples (Fig. 2d-f). Nevertheless, the expression of DNMT3b remains negative in all three groups (Fig. 2g-i).

### Three scenarios for *LKB1* promoter methylation in PJS polyps

**Fig. 3:**
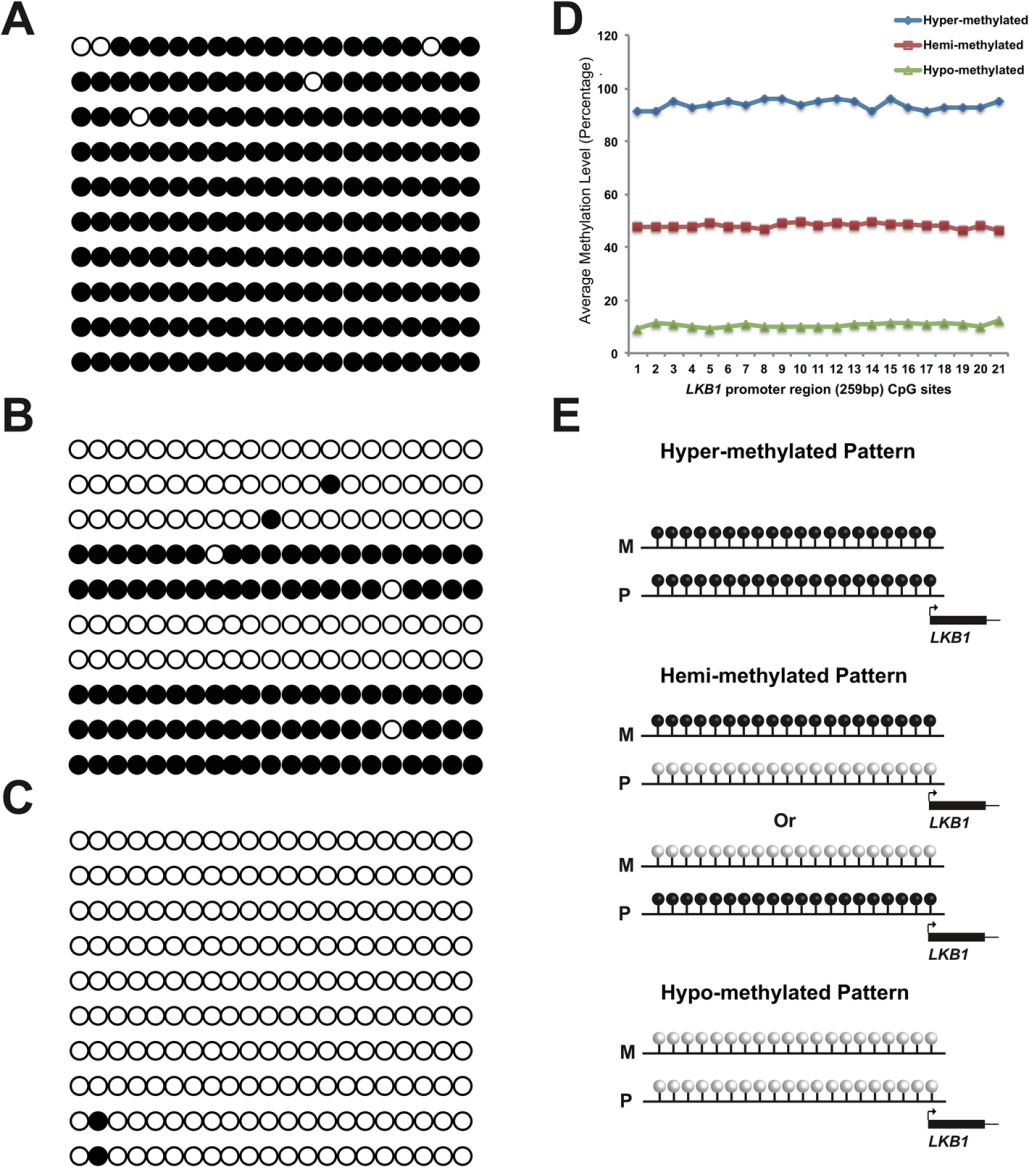
Distinct promoter methylation patterns of *LKB1* in hamartomatous polyps of PJS patients. **a**-**c** Representative of three methylation patterns of *LKB1* promoter region presented by lollipop graph. **a**: hyper-methylated pattern; **b**: hemi-methylated pattern; **c**: hypo-methylated pattern. **d** Linear plot for the average methylation level of *LKB1* promoter region for all the above patterns. **e** Diagram of three possible scenarios for different methylation patterns. M = maternal allele, P = paternal allele

In addition to the differential methylation levels, we found three methylation patterns of *LKB1* promoter in PJS polyps. We categorized average methylation rate > 75% as hyper-methylation pattern, between 25 and 75% as hemi-methylation pattern, and < 25% as hypo-methylation pattern. Among the 50 PJS polyps, 9 were hyper-methylated in *LKB1* promoter region, 37 were hemi-methylated and 14 were hypo-methylated (Fig. 3a, b). Intriguingly, the methylation within one read generally follows the all or none rule, i.e. the read is either methylated on all 21 CpGs, or unmethylated for almost all of them. These patterns are usually seen in allelic methylated regions such as imprinting genes or random allelic methylated regions as described in previous researches [13]. Thus, *LKB1* promoter methylation could be concluded into three scenarios. For hyper-methylated pattern, both paternal and maternal alleles were methylated. For hemi-methylated pattern, either paternal or maternal allele was methylated. And for hypo-methylated pattern, none of those two alleles were methylated (Fig. 3c).

### *LKB1* promoter hypo-methylation is risk factor of malignancies in PJS patients

To elucidate the possible impact of methylation level on PJS patients, serial statistical analysis was performed. We tested the average methylation level, age, sex, family history, *LKB1* germline mutation, and location between the PJS and control groups. And two factors: average *LKB1* methylation level and age are statistically significant.

Further more, PJS groups was divided into two groups by the occurrence of malignancies, and all of the above factors were analyzed by Logistic regression method to seek potential risk factors. The Logistic regression was performed under forward conditional method, and only *LKB1* promoter methylation level remains in the equation. The OR value is 0.954 and *P* < 0.05 (Table 1). Thus, hypomethylated *LKB1* promoter might act as a risk factor in the gastrointestinal malignancies of PJS patients.

**Table 1:** Evaluation of the relationship between *LKB1* promoter methylation level and gastrointestinal malignancies in PJS by Logistic regression

Variant	β	S.E	Wald	OR^a^	95% CI	*P* value
*LKB1* promoter methylation level	−0.47	0.20	5.467	0.954	0.918–0.992	0.038

^a^ OR was adjusted by age, gender, family history, *LKB1* germline mutation, and polyp locations through forward conditional method

## Discussion

Previous publications have proved the relationship between *LKB1* germline mutation and PJS [1, 7–9, 14]. In most of the PJS patients, *LKB1* exon mutation could be detected either through PCR based Sanger sequencing [6] or multiplex ligation-dependent probe amplification (MLPA) [15], or even whole exome sequencing [16]. The published mutation rate varies from 66 to 94% [17]. While in our study, *LKB1* germline mutation rate is 72%. These mutations were believed to interfere *LKB1* protein expression and/or function and further disturbed the downstream signal such as MAPK, mTOR, etc. [18]. Nevertheless, researchers also found abnormal methylated CpGs in *LKB1* promoter region by methylation specific PCR (MSP) method [11]. But since MSP could only detect one or two CpGs at the same time, it is quite difficult to fully evaluate the methylation status of *LKB1* promoter, which contains hundreds of CpGs. Other researches indicated that in “normal” crypt of colon from PJS patients, the DNA methylation pattern of cardiac-specific homeobox (*CSX)* gene is altered and might be related to the protracted clonal evolution in the crypt [12]. All these data suggested abberrant DNA methylation is involved in PJS development.

The overall methylation level in PJS patients is significantly elevated compared to the control group according to the data in this study. Our data together with previous publications has proved the involvement of *LKB1* promoter methylation in PJS polyps’ development [19]. Furthermore, we discovered distinctive methylation patterns in PJS polyps. Each represents a scenario that might explain how the hamartomatous polyps were developed. Bi-allelic methylation of *LKB1* could silence gene expression through prevention by the binding of transcription factors. While, monoallelic methylation of *LKB1* could act as secondary “strike”, as loss of heterozygosity at *LKB1* locus is quite common in PJS patients [20]. However, the role of hypomethylaiton in the development of PJS polys is still not quite clear. And the heterogeneity of *LKB1* promoter methylation status suggested it might be a potential factor to further categorizes PJS patients into groups. Thus, we have tested whether *LKB1* promoter methylation levels are co-related to the basic characteristics and prognosis of PJS patients. The results indicated that *LKB1* promoter hypo-methylation is the risk factor for malignancies among PJS patients. Although the downstream mechanism remains to be elucidated, such data might help to predict the prognosis of PJS and provide us a potential prognostic marker for clinical application.

Currently, PJS patients were recommended to take enteroscopy for every 1–3 years starting at 8–10 years [21, 22]. These examinations have increased the expenditure and reduced the quality of life for PJS patients. *LKB1* promoter methylation examination might be a more effective tool to predict the occurrence of malignant gastrointestinal cancer. Nevertheless, more efforts are required to fully evaluate the diagnostic value of *LKB1* promoter methylation.

## Conclusion

In this study, the methylation status of *LKB1* promoter region in PJS and control group was determined by bisulfite PCR and Sanger sequencing. The comparison between the two groups proved methylation level of PJS polyps is elevated in general. In addition, three distinct methylation patterns in PJS polyps were described. The identification of these patterns enables us to further categorize PJS patients into groups. More importantly, we have discovered lower DNA methylation level in this region has suggested greater chance to suffer from malignant tumors in PJS patients. Altogether, these data might contribute to the prediction of GI malignancis in PJS patients, and add an alternative tool with the current surveillance strategy.

## Material and methods

The purpose of this study was to compare PJS polyp and normal mucosa from their DNMTs expression and *LKB1* promoter methylation status, aiming at exploring the role of DNMTs in *LKB1* promoter methylation.

### Patients and sample collection

The PJS patients included in this study comprises 50 patients with DBE polypectomy from 2015 to 2018 in our hospital (Table 1). For each case, PJS is diagnosed by WHO criteria (any one of below): ≥3 hamartomatous polyps; or ≥ 1 hamartomatous polyps if family history of Peutz-Jeghers Syndrome (PJS); or prominent mucocutaneous melanosis if family history of PJS; or prominent mucocutaneous melanosis and ≥ 1 hamartomatous polyp. Only FFPE tissues from patients met the above criteria were selected for DNA extraction. As for control samples, colonoscopy biopsies were taken from routine physical examination of 50 healthy adults. The general information for patients enrolled is detailed in Table 2.

**Table 2:** General characteristic of PJS and control groups used in this study

Characteristics	PJS cases (*N* = 50) ^a^	Control (*N* = 50) ^a^
**Age (years)**		
≤ 20	16 (32%)	2 (4%)
21–30	15 (30%)	8 (16%)
31–50	18 (36%)	22 (44%)
> 50	1 (2%)	18 (36%)
**Gender**		
Female	20 (40%)	15 (30%)
Male	30 (60%)	35 (70%)
**Polyp/biopsy location**		
Stomach	2 (4%)	0
Intestine	32 (64%)	6 (12%)
Colon	16 (32%)	44 (88%)
**GI Malignancies**		
Yes	8 (16%)	0
No	42 (84%)	50 (100%)
**Family History**		
Yes	27 (54%)	0
No	23 (46%)	50 (100%)
***LKB1*** **germline mutation**		
Yes	26 (72.2%)	0
No	10 (27.8%)	50 (100%)

^a^ Data were presented in number (percentage). Total number may not be equal to the total of cases or controls due to missing or unknown data

### DNA extraction and bisulfite treatment

Genomic DNA was extracted using FFPE Tissue Genomic DNA Kit (Hooseen bio) following manufacturer’s instructions. Briefly, the FFPE tissue was cut into slices, and incubated with GA buffer in 90 °C water bath for 30 min. Centrifuge at 12000 rpm for 2 min, discard the paraffin layer and transfer the residue to a new tube. Add 25 μl proteinase K and incubated in 55 °C water bath until the tissue is fully dissolved. Transfer the supernatant and mix with GB buffer, incubate in 70 °C for 10 min. Add 250 μl ethanol, vortex and transfer to DNA conjugation column. Centrifuge at 12000 rpm for 30s. Discard the residue and wash with GD buffer twice and elute with 40 μl EB buffer. DNA was stored at − 20 °C. Meanwhile, genomic DNA of these PJS patient was also extracted from whole blood cells as previously reported.

Bisulfite treatment was performed through EZ DNA Methylation-Lightning Kit (Zymo research). Briefly, 1 μg of genomic DNA was added to 130 μl Lightning conversion reagent, incubated at 98 °C for 8 min and then 54 °C for 60 min. The mixture was then loaded to column with 600 μl M-binding buffer. Centrifuge at 12000 rpm, and wash with 100 μl M-wash buffer. After 20 min incubation with 200 μl L-Desulphonation buffer, centrifuge at 12000 rpm and wash the column twice with 200 μl M-wash buffer. Discard all residues and elute with 10 μl EB buffer. Bisulfite treated DNA was stored at − 20 °C.

### *LKB1* germline mutation detection

PCR primer of all *LKB1* exons and reaction set up was according to previous published literature [23]. The PCR product was loaded to 2% agarose gel and purified by TIANgel Mini Purification Kit (TIAGEN), and then sent for Sanger sequencing (Sangon Biotech). The result was aligned with reference genomic sequence of *LKB1* (GRCh37.p13) and all SNPs were excluded through crosscheck with NCBI SNP database.

### Immunohistochemistry of DNMT1, 3a and 3b

The FFPE tissues were cut with 4 μm slides, and emerged in xylene to remove the paraffin and followed by graded ethanol. Heat-induced epitope retrieval was conducted in EDTA solution (pH 9.0). Endogenous peroxidase was blocked by 3% H_2_O_2_ for 10 min. Rinse the slides with PBS and incubate with primary antibody (DNMT1: CatNo. 39,204, mouse monoclonal, Active Motif, dilution 1:200; DNMT3a: CatNo. ab13888, mouse monoclonal, Abcam, dilution 1:200; DNMT3b: CatNo. ab2851, rabbit polyclonal, Abcam, dilution 1:200) for 45 min. After PBS rinse, secondary antibody (REAL EnVision Detection System, Rabbit/Mouse, CatNo. K5007, DAKO) was incubated for 20 min and rinse again with PBS. Positive staining was developed by DAB for 3-5 min and slides were emerged in graded ethanol and xylene eventually sealed with cover slides.

### *LKB1* promoter methylation analysis

Bisulfite treated DNA and KAPA HiFi HotStart Uracil + ReadyMix PCR Kit (KAPA biosystems) was used to set up the system for amplification. The bisulfite PCR primers for *LKB1* promoter were designed on MethPrimer website (Fig. 1e) [24]. The primer sequences are listed as follow: Forward 5′- GAG GAT GAT TTA GTA TTG AAA AGT-3′; Reverse 5′- AAC AAC AAA AAC CCC AAA AA-3′, product size: 259 bp (containing 21CpG sites). The reaction was performed under 95 °C for 5 min, followed with 39 cycles of 98 °C for 20s, 59 °C for 15 s, and 72 °C for 1 min; and then 72 °C for 10 min. The product was uploaded to 1.5% agarose gel and the purification was done by TIANgel Mini Purification Kit (TIANGEN). The purified product was ligated to pGM-Simple-T Fast Vector (TIANGEN) by T4 DNA ligase (NEB). The ligated vector was transfected into DH5α competent cells. LB agar plate was used for monoclonal selection. Sanger sequencing was sent to Sangon Biotech. Each sample was required at least 10x coverage. Sequences was aligned to reference *LKB1* promoter sequence, and visualized by BiQ analyzer [25].

### Statistical analysis

The SPSS 22.0 software was used for statistical analysis. The comparison between PJS and control group on *LKB1* methylation levels, age, sex, family history, *LKB1* germline mutation, and polyp location was performed by Kruskal Wallis Test. Odds ratio (OR) was calculated by logistic regression (forward conditional method) to evaluate the association between methylation levels of *LKB1* with the risk for gastrointestinal malignancies in PJS patients, adjusting for age, sex, polyp location, family history, and *LKB1* germline mutation. *P* < 0.05 is considered statistically significant.

## Supplementary information

**Additional file 1.**

## Data Availability

The major data sets supporting the results of this article are included within the article and its additional files.

## Abbreviations

LKB1: Liver Kinase B1
PJS: Peutz-Jeghers Syndrome
DNA: Deoxyribonucleic Acid
DNMT: DNA Methyltransferase
DBE: Double balloon pushed enteroscopy
PCR: Polymerase Chain Reaction
WHO: World Health Organization
FFPE: Formalin-Fixed and Paraffin-Embedded
GI: Gastrointestinal
NCBI: National Center for Biotechnology Information
SNP: Single nucleotide polymorphism
EDTA: Ethylene Diamine Tetraacetic Acid
PBS: Phosphate Buffered Saline
DAB: Diaminobenzidine
LB: Lysogeny broth
OR: Odds ratio
HE: Hematoxylin eosin
SEM: Standard error for mean
S.E: Standard error
CI: Confidence interval
MLPA: Multiplex ligation-dependent probe amplification
MSP: Methylation specific polymerase chain reaction
CSX: Cardiac-specific homeobox
